# Association of hospital nursing and postsurgical sepsis

**DOI:** 10.1371/journal.pone.0258787

**Published:** 2021-10-18

**Authors:** Andrew M. Dierkes, Linda H. Aiken, Douglas M. Sloane, Matthew D. McHugh

**Affiliations:** 1 Department of Acute and Tertiary Care, School of Nursing, University of Pittsburgh, Pittsburgh, Pennsylvania, United States of America; 2 Center for Health Outcomes and Policy Research, School of Nursing, University of Pennsylvania, Philadelphia, Pennsylvania, United States of America; Heidelberg University Hospital, GERMANY

## Abstract

Despite concerted research and clinical efforts, sepsis remains a common, costly, and often fatal occurrence. Little evidence exists for the relationship between institutional nursing resources and the incidence and outcomes of sepsis after surgery. The objective of this study was to examine whether hospital nursing resource quality is associated with postsurgical sepsis incidence and survival. This cross-sectional, secondary data analysis used registered nurses’ reports on hospital nursing resources—staffing, education, and work environment—and multivariate logistic regressions to model their association with risk-adjusted postsurgical sepsis and mortality in 568 hospitals across four states. Better work environment quality was associated with lower odds of sepsis. While the likelihood of death among septic patients was nearly seven times that of non-septic patients, better nursing resources were associated with reduced mortality for all patients. Whereas the preponderance of sepsis research has focused on clinical interventions to prevent and treat sepsis, this study describes organizational characteristics hospital administrators may modify through organizational change targeting nurse staffing, education, and work environments to improve patient outcomes.

## Introduction

Sepsis is common, costly, and often fatal. Each year, U.S. hospitals spend over $20 billion managing more than 1 million septic patients [1]. The aggregated costs exceed that of other hospital in-patients, but, despite this investment, sepsis remains a leading cause of in-hospital death [2]. Serious international efforts to advance and standardize sepsis recognition and treatment have produced clinical guidelines, such as the *Surviving Sepsis Campaign* [3], and process measures, such as the National Quality Forum-endorsed Early Management Bundle for Severe Sepsis/Septic Shock (SEP-1). Despite these efforts, hospital-level measures of sepsis incidence and mortality vary significantly by institution even after controlling for patient characteristics [4, 5].

Bedside nurses are at the forefront of sepsis surveillance, prevention, and early recognition/response [6]. They monitor vital signs, advise medical practitioners of changes in a patient’s condition, and implement orders to draw labs (including blood cultures and lactate levels), administer antibiotics, and resuscitate patients with intravenous fluids, all of which are time-sensitive sepsis interventions. Hospital nursing resources, including staffing [7], education [8], and the work environment [9], have been shown repeatedly to help explain facility-level variation in hospital infection and complication rates [10, 11], as well as measures of mortality and failure to rescue [12–15]. Little is known about the impact of nurse staffing, education, and the work environment on sepsis incidence and mortality, but these resources may represent key administrative and policy interventions to achieve better outcomes at lower costs. The objective of this study was to document the association between these nursing resources and surgical patient sepsis and mortality.

## Methods

### Design and data

We conducted a cross-sectional analysis of secondary data from three sources: 1) state inpatient discharge abstracts from California, Florida, Pennsylvania, and New Jersey, 2) the 2006 Multi-State Nursing Care & Patient Safety Study survey of RNs, and 3) the 2006 American Hospital Association (AHA) Annual Survey. RN recipients of the Multi-State Nursing Care & Patient Safety Study survey were notified in writing using materials approved by the University of Pennsylvania institutional review board (IRB) that participation was voluntary and by completing the survey, they were giving consent to participate. The survey and consent materials included study team contact information for survey recipients to ask questions and discuss any issues. The AHA survey data is secondary administrative data on hospitals acquired under a data use agreement with the AHA. The patient discharge abstracts are secondary deidentified billing data acquired under a data use agreement with each state agency. We did not recruit (or consent) participants for this study as we used secondary data sources. The protocol for this study was authorized for review exemption by the University of Pennsylvania IRB.

### Sample

#### Hospitals

We examined data from 568 non-federal general acute care hospitals across California (n = 218), Florida (n = 147), Pennsylvania (n = 131), and New Jersey (n = 72).

#### Nurses

Information about hospital nursing resources came from the 2006 Multi-State Nursing Care & Patient Safety Study. This survey was sent to a large random sample of RNs. Nurse reports of their work environments, workloads, and education were aggregated within each facility where respondents identified working to derive hospital-level measures of nursing resources. We obtained responses from >70% of hospitals in the four states. The average hospital in the final analytical dataset had 60 nurse respondents. Additional details regarding the survey methodology and representation of hospitals and patients achieved are described elsewhere [16].

#### Patients

Inpatient discharge abstracts for patients cared for across all 568 study hospitals were obtained from their respective states. Patients from CA, PA, and NJ were hospitalized in 2005 and 2006. Florida patients were hospitalized in 2006 and 2007 to reflect a year delay in administering the Florida nurse survey. The final analytical dataset included 1,241,330 general, orthopedic, and vascular surgery patients ages 19–89 with lengths of stay >1 day and with complete information on all independent and dependent variables. The diagnosis related groups used to identify these patients are listed in Table 1.

**Table 1:** Surgical patient diagnosis related group codes.

**General**
146, 147, 148, 149, 150, 151, 152, 153, 154, 155, 157, 158, 159, 160, 161, 162, 164, 165, 166, 167, 170, 171, 191, 192, 193, 194, 195, 196, 197, 198, 199, 200, 201, 257, 258, 259, 260, 261, 262, 263, 264, 265, 266, 267, 268, 285, 286, 287, 288, 289, 290, 291, 292, 293, 493, 494
**Orthopedic**
209, 210, 211, 213, 216, 217, 218, 219, 223, 224, 225, 226, 227, 228, 229, 230, 232, 233, 234, 471, 491, 496, 497, 498, 499, 500, 501, 502, 503, 519, 520, 537, 538, 544, 545, 546
**Vascular**
110, 111, 113, 114, 119, 120

### Measures

#### Hospital-level measures

Nurse staffing represented the mean patient-to-nurse ratio by hospital. Responses from nurses working on units where staffing is atypical (specifically: the emergency room, psychiatric, labor/delivery units, and outpatient settings) were excluded from the staffing metric, but still used to inform measures of other nursing resources. Nurse education represented the proportion of RNs by hospital with a baccalaureate nursing or higher degree. Nurse responses to the 31-item Practice Environment Scale of the Nursing Work Index (PES-NWI) [17] were averaged first within and then across each of 4 subscales to generate a global, facility-level measure of the nurse work environment. These subscales assessed “Nurse Participation in Hospital Affairs”, “Nursing Foundations for Quality of Care”, Nurse Manager Ability, Leadership, and Support of Nurses”, and “Collegial Nurse–Physician Relations”. The “Staffing and Resource Adequacy” subscale was excluded from the measure due to its conceptual overlap with our direct measure of staffing. The Nursing Work Index from which the Practice Environment Scale is derived consists of organizational attributes characteristic of Magnet^®^ hospitals, which are known to achieve high quality care through excellence in nursing [17].

Four additional hospital characteristics derived from the 2006 American Hospital Association (AHA) Annual Survey served as control variables: state, size, teaching status, and technology status. “State” reflected the geographic location of each hospital (CA, FL, PA, or NJ). The number of licensed hospital beds in each institution determined its size: small (≤100 beds), medium (101–250 beds), or large (>250 beds). Teaching status reflected the ratio of resident physicians and fellows to hospital beds: nonteaching (no postgraduate trainees), minor teaching (≤1:4 ratio), and major teaching (>1:4 ratio). Hospitals with facilities for open-heart surgery and/or major organ transplants were considered “high-technology”. “Low-technology” hospitals lacked facilities for both procedure types. Hospital controls also included two variables representing the proportion of nurse respondents reporting from 1) medical-surgical and 2) intensive care units.

#### Patient measures

Sepsis cases and the 27 comorbidities used to risk adjust, were identified using *International Classification of Diseases*, *Ninth Revision*, *Clinical Modification (ICD-9-CM)* codes as established by Silber et al. [18] and Elixhauser et al. [19], respectively. Our mortality variable indicated in- or out-of-hospital death within 30 days of admission. Additional risk adjustment variables were patient age and sex, an indicator for patients transferred to the hospital, as well as 61 dummy variables for surgical procedure.

### Data analysis

Descriptive statistics provide information about 1) characteristics of the 568 study hospitals, 2) the numbers and percentages of septic and non-septic surgical patients in hospitals with different characteristics, including nursing resources, and 3) the characteristics of septic and non-septic patients, including mortality, demographics, surgery type, and comorbidities. Logistic regression models estimated the odds of surgical patients developing sepsis, and the odds of septic and non-septic patients dying, in hospitals with different nursing resources, before and after controlling for other patient and hospital characteristics. For these regression analyses, we scaled the nurse education variable so that a one-unit increase represented 10% more nurses with at least a BSN. Hospital work environment categories were derived from average hospital-level PES-NWI scores. Grouping hospitals by quartile and merging the middle two quartiles produced a measure which contrasted hospitals with the “worst” (first quartile), “mixed” (second and third quartiles), and “best” (fourth quartile) environments. These ordinal categories were coded 0, 1, and 2, respectively, which imposed a linear relationship on differences between categories. Each one-unit difference in nurse staffing represented a change in the average workload of one patient per nurse.

Of the 1,647,498 surgical patients cared for in hospitals for which we had information on nursing resources and structural characteristics, we excluded 102,130 for ages >89 or <19, 303,281 for length of stay ≤1 day, 1 observation that represented a second admission for a patient already represented within the study period, 29 for missing or incomplete information on sex, 550 for missing information on transfer status, and 177 for DRGs 156 ("Stomach, Esophageal & Duodenal Procedures Age 0–17") and 231 ("Unused DRG Placeholder Since 10/1/03"), which did not apply to this study population. There was no missing data among both independent and dependent variables for subjects in the final analytical dataset. All analyses controlled for patient and hospital characteristics and used robust estimation procedures to account for the clustering of patients within hospitals and were performed using Stata version 15.1 (Stata Corp, College Station, TX). Tests were two-sided and P < .05 was the threshold level of significance.

## Results

### Characteristics of hospitals and patients

Table 2 shows characteristics of the study hospitals, including the three nursing resource measures, and the distribution of surgical patients overall and of septic and non-septic subgroups across these hospitals. The column headings show that there were 1.24 million surgical patients in the 568 study hospitals, and that 27,524 (2.2%) of them were septic. In 48% of hospitals, the average nurse-to-patient ratio was less than 5:1. The nurses in these better-staffed hospitals cared for 52% of all surgical patients and 53% of surgical sepsis patients. Three-fourths of the hospitals had less than 50% BSN-prepared nurses, and the nurses in these hospitals cared for 67% of all surgical patients and 66% of surgical sepsis patients. Hospitals were distributed across “worst” (23%), “mixed” (52%), and “best” (25%) work environments. Less than 30% of all groups of surgical patients were cared for in hospitals with “best” work environments.

**Table 2:** Study hospitals and surgical patients.

	No. (%)				
Hospital Characteristic	Hospitals (n = 568)	Surgical Patients			
		All (n = 1,241,330)	Septic (n = 27,524)	Non-Septic (n = 1,213,806)	P Value^a^
**Staffing**					
<4	100 (18)	212,609 (17)	5,172 (19)	207,437 (17)	< .001
4-<5	169 (30)	431,440 (35)	9,425 (34)	422,015 (35)	
5-<6	163 (29)	373,407 (30)	8,065 (29)	365,342 (30)	
6-<7	70 (12)	134,362 (11)	3,051 (11)	131,311 (11)	
≥7	66 (12)	89,512 (7)	1,811 (7)	87,701 (7)	
**Education**					
0-<20%	30 (5)	35,057 (3)	701 (3)	34,356 (3)	< .001
20-<30%	73 (13)	119,602 (10)	2,360 (9)	117,242 (10)	
30-<40%	152 (27)	289,007 (23)	6,458 (23)	282,549 (23)	
40-<50%	169 (30)	379,142 (31)	8,550 (31)	370,592 (31)	
50-<60%	98 (17)	282,267 (23)	6,363 (23)	275,904 (23)	
≥60	46 (8)	136,255 (11)	3,092 (11)	133,163 (11)	
**Work Environment** ^**b**^					
Worst	133 (23)	216,799 (17)	4,951 (18)	211,848 (17)	.001
Mixed	295 (52)	659,204 (53)	14,744 (54)	644,460 (53)	
Best	140 (25)	365,327 (29)	7,829 (28)	357,498 (29)	
**State**					
California	218 (38)	520,281 (42)	12,265 (45)	508,016 (42)	< .001
Florida	147 (26)	273,096 (22)	5,667 (21)	267,429 (22)	
Pennsylvania	131 (23)	305,513 (25)	5,387 (20)	300,126 (25)	
New Jersey	72 (13)	142,440 (11)	4,205 (15)	138,235 (11)	
**Bed Size (number of beds)**					
Small (≤100)	58 (10)	45,057 (4)	856 (3)	44,201 (4)	< .001
Medium (101–250)	250 (44)	371,195 (30)	8,306 (30)	362,889 (30)	
Large (≥250)	260 (46)	825,078 (66)	18,362 (67)	806,716 (66)	
**Teaching Status**					
Non-teaching	286 (50)	534,302 (43)	11,972 (43)	522,330 (43)	.07
Minor teaching	237 (42)	519,719 (42)	11,526 (42)	508,193 (42)	
Major teaching	45 (8)	187,309 (15)	4,026 (15)	183,283 (15)	
**Technology Status**					
High-tech	255 (45)	781,058 (63)	17,344 (63)	763,714 (63)	.75

^a^Tests of significance are Pearson Chi2 analyses.

^b^Work environment quality level categories were derived from quartile groupings of hospitals based on mean hospital-level PES-NWI scores. The middle two quartiles were merged to produce a measure that contrasted hospitals with “worst” (first quartile), “mixed” (second and third quartiles), and “best” (fourth quartile) work environments.

The distribution of hospitals and patients across the four states reflected the size of the states. Hospitals were evenly divided between teaching and non-teaching hospitals, and by high- and low-technology status. Most teaching hospitals were “minor teaching” institutions. Roughly half (46%) had 250 or more beds, while only 10% had 100 beds or fewer. Surgical patients were similarly distributed with few exceptions. The percentages of surgical patients and surgical sepsis patients were disproportionately high in California and in high-tech hospitals.

As shown in row one of Table 3, deaths were decidedly more likely among septic patients (23%) than other surgical patients (2%). This rate varied substantially by surgical group. While orthopedic surgery cases comprised 52% of all patients, they accounted for only 19% of septic patients. Conversely, only 1 in every 20 surgical patients underwent a vascular procedure, but these patients represented 21% of septic cases. Similarly, general surgery patients were 43% of the total surgical population but made up 60% of sepsis cases. On average, septic patients had twice the number of comorbidities compared to non-septic surgical patients (3.6 vs. 1.8). Septic patients had substantially higher rates of virtually all comorbidities; hypertension and congestive heart failure were most common and present in more than a quarter of all septic patients. The patient characteristics shown in the table also indicate that sepsis occurred less often among women than men and involved somewhat older patients.

**Table 3:** Characteristics of surgical and surgical sepsis patients.

	No. (%)			
Characteristic	All (n = 1,241,330)	Septic (n = 27,524)	Non-Septic (n = 1,213,806)	P Value^a^
Deaths within 30 days of admission	26,474 (2)	6,265 (23)	20,209 (2)	< .001
Female	704,838 (57)	12,751 (46)	692,087 (57)	< .001
Age, mean (Standard Deviation)	60.6 (17.3)	65.7 (15.5)	60.5 (17.3)	< .001
**Surgical Groups**				
General surgery	533,064 (43)	16,635 (60)	516,429 (43)	< .001
Orthopedic surgery	641,675 (52)	5,093 (19)	636,582 (52)	
Vascular surgery	66,591 (5)	5,796 (21)	60,795 (5)	
**Comorbidities** ^**b**^				
Number of comorbidities, mean (SD)	1.9 (1.7)	3.6 (2.2)	1.8 (1.6)	< .001
Comorbidity				
Hypertension	606,229 (49)	13,671 (50)	592,809 (49)	.005
Congestive Heart Failure	90,904 (7)	7,228 (26)	83,676 (7)	< .001
Renal Failure	68,499 (6)	6,973 (25)	61,526 (5)	< .001
Chronic Pulmonary Disease	192,774 (16)	6,272 (23)	186,502 (15)	< .001
Diabetes, Uncomplicated	190,180 (15)	4,813 (17)	185,367 (15)	< .001
Weight Loss	25,115 (2)	4,133 (15)	20,982 (2)	< .001
Diabetes, Complicated	43,428 (4)	2,982 (11)	40,446 (3)	< .001
Peripheral Vascular Disorders	53,231 (4)	2,789 (10)	50,442 (4)	< .001
Liver Disease	36,006 (3)	2,563 (9)	33,443 (3)	< .001
Other Neurological Disorders	41,360 (3)	2,540 (9)	38,820 (3)	< .001
Valvular Disease	66,298 (5)	2,443 (9)	63,855 (5)	< .001
Metastatic Cancer	45,400 (4)	2,223 (8)	43,177 (4)	< .001
Obesity	109,254 (9)	1,977 (7)	107,277 (9)	< .001
Depression	99,342 (8)	1,967 (7)	97,375 (8)	< .001
Hypothyroidism	120,710 (10)	1,961 (7)	118,749 (10)	< .001
Alcohol Abuse	31,072 (3)	1,405 (5)	29,667 (2)	< .001
Solid Tumor Without Metastasis	25,440 (2)	1,348 (5)	24,092 (2)	< .001
Blood Loss Anemia	19,477 (2)	1,084 (4)	18,393 (2)	< .001
Pulmonary Circulation Disorders	16,732 (1)	1,037 (4)	15,695 (1)	< .001
Drug Abuse	23,168 (2)	1,011 (4)	22,157 (2)	< .001

^a^Pearson Chi-square analyses for categorical variables. Two-sample t test for continuous variables.

^b^Rheumatoid Arthritis/Collagen Vascular, Deficiency Anemia, Paralysis, Psychoses, Peptic Ulcer Disease Excluding Bleeding, Lymphoma, and AIDS/HIV were included in the analysis, but omitted from this table for brevity as <3% of all surgical patients and surgical sepsis patients exhibited these comorbidities.

#### Nursing resources and sepsis

Table 4 displays the unadjusted and adjusted odds ratios (ORs) representing the change in odds of developing sepsis (Panel A) and the odds of dying (Panels B and C) associated with a one-unit increase in each of the nursing resources studied: staffing, education, and the work environment. The fully adjusted models (those including patient and hospital characteristics) in Panel A show that only differences in the work environment were significantly related to the odds of sepsis. For each 1-unit difference in the quality of the work environment (e.g., from “worst” to “mixed” or “mixed” to “best”), patients experienced 6% lower odds of developing sepsis (OR, 0.94; 95% CI, 0.90–0.99; P = 0.01). This implies that the odds of surgical patients developing sepsis while cared for in “best” work environments are lower than for patients in “worst” work environments by a factor of 0.88 (i.e., 0.94^2), or 12% lower.

**Table 4:** The impact of nursing resources on odds of sepsis and death.

	Bivariate		Fully Adjusted^a^	
Resource	OR (95% CI)	P Value	OR (95% CI)	P Value
**A. Odds of Sepsis**				
Staffing	0.98 (0.95–1.01)	.15	1.02 (1.00–1.05)	.08
Education	1.03 (1.00–1.06)	.08	1.02 (0.99–1.04)	.20
Work Environment	0.97 (0.91–1.03)	.26	0.94 (0.90–0.99)	.01
**B. ODDS OF DEATH (from one fully adjusted model without interactions)**				
Staffing	1.04 (1.02–1.07)	< .001	1.02 (1.00–1.04)	.05
Education	0.93 (0.91–0.95)	< .001	0.94 (0.92–0.96)	< .001
Work Environment	0.92 (0.87–0.96)	< .001	0.94 (0.91–0.98)	< .003
Sepsis	17.41 (16.69–18.15)	< .001	6.57 (6.26–6.89)	< .001
**C. ODDS OF DEATH (from three fully adjusted models, each with one interaction)** ^**b**^				
Sepsis	-	-	5.71 (4.69–6.94)	< .001
Staffing x Sepsis	-	-	1.03 (0.99–1.07)	.15
Sepsis	-	-	7.13 (5.94–8.56)	< .001
Education x Sepsis	-	-	0.98 (0.94–1.02)	0.38
Sepsis	-	-	6.65 (6.10–7.25)	< .001
Work. Environment x Sepsis	-	-	0.99 (0.93–1.05)	0.73

^a^Adjusted models include patient and hospital characteristics (patient n = 1,241,330, hospital n = 568).

Patient characteristics: age, sex, transfer, 27 Elixhauser comorbidities (excluding fluid & electrolyte d/o, coagulopathy, AND cardiac arrhythmias; uncomplicated/complicated hypertension combined into one indicator); and 61 surgical procedures.

Hospital characteristics: size, technology status, teaching status, hospital state, and an indicator for proportion of nurse survey respondents reporting from intensive care and medical-surgical units.

^b^The adjusted effect of each nursing resource for the fully adjusted models presented in Panel C was the same as in the main-effects model shown in Panel B.

#### Nursing resources and patient mortality

Panels B and C of Table 4 present odds ratios from logistic regression models that estimate the impact of nursing resources and sepsis on 30-day mortality among surgical patients. In the main effects models (those excluding interactions) each of these variables—sepsis and each of three nursing resources—were associated with odds of death before and after adjusting for patient and hospital characteristics. In the fully adjusted model, septic patients experienced 6.6 times the odds of death relative to their non-septic counterparts (OR, 6.57; 95% CI, 6.26–6.89; P < .001). Each additional patient per nurse was associated with a 2% increase in odds of death (OR, 1.02; 95% CI, 1.00–1.04, P = .05). While this effect may seem small, it implies that patients in hospitals where the average patient-to-nurse ratio is 7:1 rather than 4:1 would have 6% higher odds of dying (i.e., 1.02^ 3 = 1.06). A 1-unit increase in education (representing a 10% increase in BSN-prepared nurses) was associated with 6% lower odds of death among postsurgical sepsis patients (OR, 0.94; 95% CI, 0.92–0.96; P < .001). In three-quarters of the hospitals studied, more than half of the nursing workforce lacked a BSN. Even the best of these hospitals fell short of the Institute of Medicine’s (IOM’s) current 80% BSN-prepared benchmark (Institute of Medicine, 2011) by 30%. Based on this study, that 30% change would lower the odds of death among surgical sepsis patients in that hospital by a factor of 0.83 (i.e., 0.94^3) or 17% lower. Just as the work environment was associated with the odds on sepsis, so too was each unit increase in work environment quality associated with a 6% decrease in mortality (OR, 0.94; 95% CI, 0.91–0.98; P = .003).

In Panel C of Table 4 we introduce interaction terms to evaluate whether the effects of the nursing resources on odds of dying were the same for septic patients (who had a mortality rate of 23%) and non-septic patients (who had a mortality rate of 2%). We found no evidence that they were different. While the coefficient associated with the difference between septic and non-septic patients differs depending on which interaction term was included in the model (from 5.7 to 7.1), the adjusted effect of each nursing resource was the same as in the main-effects model shown in Panel B, which is why we do not reproduce them in the final panel. The more important point is that no interactions are significant. This implies that the effects of these resources in reducing deaths are the same among septic and non-septic surgical patients.

## Discussion

Bedside nurses provide direct care throughout the continuum of surgical care. Their place at the bedside and professional training position them to provide the surveillance and implement the clinical interventions that current sepsis guidelines and care bundles recommend. It is increasingly evident that nursing resources are associated with the delivery of care and, consequently, patient infection rates, mortality, and death after infection. As this study documents, patients cared for in hospitals with better work environments have lower odds of sepsis. All surgical patients, including septic cases, are better off in hospitals with better work environments, better staffing, and a higher percentage of BSN-prepared nursing staff, where they have lower odds of death. The lack of these resources should concern all providers caring for surgical and surgical sepsis patients.

Sepsis prevention reduces the need for sepsis treatment and the possibility of septic death. In large part, sepsis prevention is infection prevention, which is grounded in straightforward practices such as handwashing and use of personal protective equipment. These hygienic routines are intuitive and do not require a college degree to implement. However, the organizational context can facilitate or impede consistent adherence. This study’s findings suggest room for substantial reductions in sepsis incidence through improved work environments. The adjusted differences in work environments across hospitals (or differences between hospitals with the “worst,” “mixed,” and “best” environments) involve 6% decreases in odds of postsurgical sepsis (OR, 0.94; 95% CI, 0.90–0.99; P = .01). Based on these findings, work environment deficiencies are related to 1,269 excess sepsis cases, or 4.6% of septic patients in this study. These could have been averted if every study hospital had a “best” work environment. In addition to this excess burden of disease, sepsis represents a significant financial burden. Applying a recent estimation of $63,800 for the added cost of sepsis among general surgery patients [20] to the patients in the 428 study hospitals with suboptimal work environments, the missed savings (or avoidable expenses) over the course of the study exceed $74 million. Better nursing resources may reasonably be assumed to be associated with outcomes for many non-surgical sepsis patients and non-septic patients as well, delivering significantly more value.

While prevention is ideal, most sepsis is community acquired; these patients develop the infection without exposure to nursing care and its potential for prevention. For these, and other cases in which prevention is unsuccessful, facilitating sepsis treatment is paramount. This study found that septic patients cared for in hospitals with more BSN-prepared nurses had lower odds of death. Each 10% increase in percent BSN-prepared nursing staff was associated with a 6% decrease in odds of death (OR, 0.94; 95% CI, 0.92–0.96; P = .001). Three-quarters of hospital nursing workforces in the study were less than 50% BSN-prepared. Not a single facility had achieved the 80% goal later promoted by the Institute of Medicine [21]. Based on these findings, had every hospital in the study achieved this goal, they could have averted 804 postsurgical sepsis deaths—a 13% decrease over the course of the study. Staffing and work environment make an additional contribution above and beyond that of education. Improvements in each of these three resources are associated with diminished odds on dying, an effect that is as large for the more complex sepsis patients as for other surgical patients.

### Limitations

While this study draws strength from its considerable size, a few limitations remain. We linked patients and nurses in our data to a common hospital and studied nursing as a hospital-level resource. This is useful for workforce policymaking and reflects the reality that patients receive care from multiple nurses across different units during their hospitalization. However, our inability to link individual patients with individual nurses prevents us from accounting for variation in exposure to the nursing resources of interest that may exist among patients within the same hospital. Similarly, our data lacked direct measures of clinical interventions in response to sepsis. Future work may benefit from a panel study design examining changes in nursing resources over time and associated patient outcomes as well as from inclusion of process measures. The study’s cross-sectional design constrains conclusions to associations, the strength of which should not be ignored, but also not confused with a causal link. Finally, this study examined sepsis and mortality among a sample of general, orthopedic, and vascular surgery patients. These are common surgery types, but the results may not apply to all surgical patients or patients hospitalized for medical treatment.

## Conclusion

Clinical guidelines are necessary but insufficient to solve the enormous problem of sepsis. The context in which they are implemented likely mediates their effectiveness. Research has identified the association of hospital characteristics, including number of beds and teaching status, on postsurgical sepsis incidence and mortality [22]. However, these characteristics are difficult, if not impossible, to change. The nursing resources in this study are modifiable, giving hospital administrators actionable insight to improve patient outcomes through organizational change. Hospitals may improve their work environments by pursuing Magnet® recognition [12] and hire with a preference or requirement for BSN-prepared nursing staff. This study quantified the associations between these nursing resources and postsurgical sepsis incidence and mortality, but system-level interventions are likely to affect every patient exposed to that improved inpatient setting, translating to broader impact.

## Supporting information

S1 Data (CSV)
